# Battling COVID-19: using old weapons for a new enemy

**DOI:** 10.1186/s40794-020-00107-1

**Published:** 2020-05-20

**Authors:** Rohit Kumar, Nitin Gupta, Parul Kodan, Ankit Mittal, Manish Soneja, Naveet Wig

**Affiliations:** 1 Department of Medicine, All India Institute of Medical Sciences, New Delhi, 110029 India; 2 Department of Infectious Diseases, Kasturba Medical College, Manipal, Karnataka 576104 India; 3 Dr Ram Manohar Lohia hospital & Post-Graduate Institute of Medica education and Research, New Delhi, 110001 India

**Keywords:** SARS-CoV-2, Hydroxychloroquine, Lopinavir/ritonavir, Remdisivir, Nitazoxanide, Tocilizumab

## Abstract

Coronavirus disease-19 (COVID-19) has reached pandemic proportions. Most of the drugs that are being tried for the treatment have not been evaluated in any randomized controlled trials. The purpose of this review was to summarize the in-vitro and in-vivo efficacy of these drugs on Severe Acute Respiratory Syndrome (SARS-CoV-2) and related viruses (SARS and Middle East Respiratory Syndrome) and evaluate their potential for re-purposing them in the management of COVID-19.

## Introduction

**Fig. 1:**
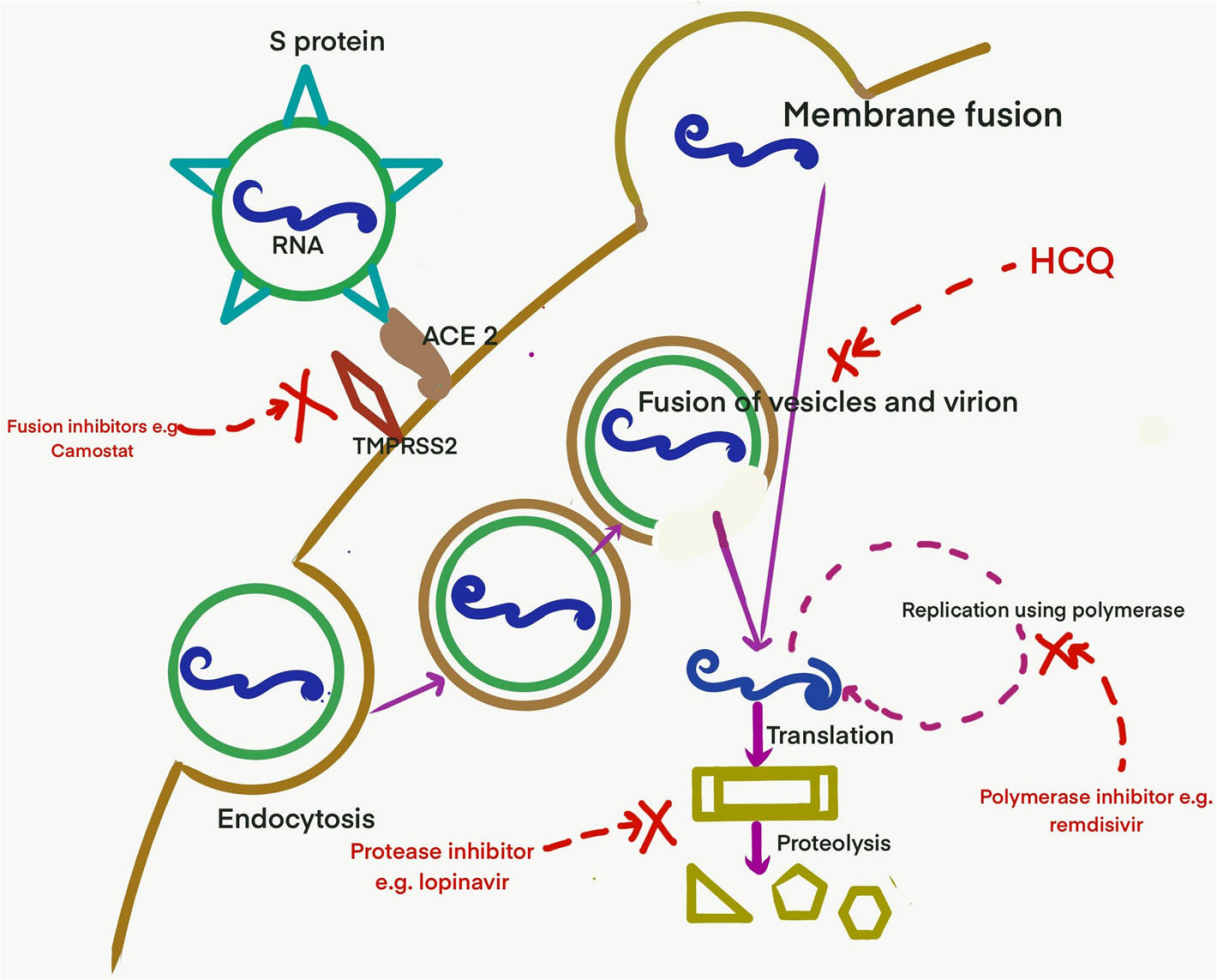
Entry and replication of SARS-CoV-2 and the drugs that inhibit the various steps

Coronavirus disease 2019 (COVID-19), a disease caused by severe acute respiratory syndrome coronavirus 2 (SARS-CoV-2), was first reported from Wuhan, China in December 2019 and it has already claimed more than forty thousand lives [1]. Although supportive measures and stringent infection control measures remain as the cornerstone of management, there is no known effective antiviral for this disease. After the spike (S) protein of the virus interacts with angiotensin-converting enzyme (ACE) receptor of the host cell, the virus enters by membrane fusion or receptor-mediated endocytosis. This is followed by replication using RNA dependent RNA polymerase, translation, virus assembly and release (Fig. 1). Several existing drugs have been identified that are postulated to act on one of these critical steps (Fig. 1). While the efforts to develop new and effective drugs are ongoing; until there are more definitive answers, effective repurposing from the existing arsenal of antivirals are being used every day. There is a call to deal with this pandemic at a war footing. Every intervention, howsoever small, with a potential benefit are being explored every day. Although, Infectious disease society of America recommends the use of the repurposed drugs in the setting of clinical trials alone due to lack of evidence; data from related viruses (like SARS-CoV-1 and MERS), in-vitro studies and growing shreds of clinical evidence from this pandemic are being used to choose the drugs which can be repurposed [2]. The drugs have been discussed under the following headings: anti-parasitic drugs, protease inhibitors, polymerase inhibitors, fusion inhibitors, monoclonal antibodies and miscellaneous (Table 1 and Table 2).

**Table 1:** Summary of clinical studies of significance of certain important drugs used for treatment of COVID-19

Study	Number of patients	Type of study	Patient population	Study arms	Results	Ref
Hydroxychoroquine						
Gautret et al., France	36	Single arm trial	All positive cases	HCQ-20, No HCQ- 16	Virological clearance on Day 6–70% in HCQ vs 12.5% in controls (*p* = 0.001)	[3]
Tang et al., China	150	Multi-centric open labelled randomized controlled trial	All positive cases	75- HCQ, 75- No HCQ	No difference in virological conversion rate at day28 (*p* = 0.341). There was no difference in improvement in clinical symptoms at day 10.	[4]
Mahevas et al., France	181	Multi-centric retrospective study	All positive cases with pneumonia	84-HCQ, 97- no HCQ	No difference in worse clinical outcomes (transfer to ICU within 7 days and/or death) between the two arms (RR- 0.93)	[5]
Magagnoli et al., USA	368	Retrospective case control study	All positive veterans	HCQ- 97, HCQ + azithromycin- 113, no HCQ- 158	Risk of death was found to be higher in those patients who received HCQ alone compared to no HCQ (*p* = 0.003)	[6]
Lopinavir/ritonavir						
Cao et al., China	199	Randomized open labelled trial	All positive patients with respiratory illness	LPV/r- 99 No LPV/r- 100	Did not show any decrease in time to clinical improvement, mortality or viral load after addition of LPV/r	[7]
Remdisivir						
Grein et al., Multinational study	53	Multi-centric single-arm study	Patients with oxygen saturation of less than 94%	No control arm	Improvement in oxygen support class was demonstrated in 68% of the patients	[8]
Tocilizumab						
Roumier et al., France	30	Case control study	Patients (< 80 years of age) with severe disease who were rapidly deteriorating	Controls matched for age and severity	Lesser ICU admission and requirement of mechanical ventilation when compared to controls (matched for age and severity)	[9]

**Table 2:** Summary of some drugs that can be repurposed for management of COVID-19

Name	Mechanism of action	In-vitro studies				In-vivo studies			
		SARS	MERS	SARS-CoV-2	Others	SARS	MERS	SARS-CoV-2	Others
Alisporivir	Cyclophilin mediated inhibition of viral replication	Completely blocked replication [10].	Inhibit cytopathic effect of virus in cell culture [10].	No studies	HCoV-229E [11], hepatitis C [12], hepatitis B [13], flaviviruses [14]	Not effective in mouse model [10]	No animal model studies	No studies	Effective in HCV
Arbidol (Umifenovir)	Intercalation into membrane lipids- inhibition of membrane fusion [15]	In-vitro effectiveness	No studies	In-vitro effectiveness	Influenza, Hepatitis C, Flaviviruses [15]	No studies	No studies	Combined arbidol and LPV/r better than LPV/r alone [16]	Prophylaxis and treatment of influenza [15]
Auranofin [17, 18]	Cellular oxidative stress and anti-inflammatory	No studies	No studies	In-vitro effective	HIV	No studies	No studies	No studies	No studies
Doxycycline	Chelation of matrix metalloproteinase [19] Anti-inflammatory	No studies	No studies	In-vitro effective [20]	Dengue, Chikungunya, Crimean Congo haemorrhagic fever, HIV	No studies	No studies	No studies	Dengue [21]
Isoprinosine or Inosine-pranobex	Immunomodulatory drug with antiviral activity [22, 23]	No studies	No studies	No studies	Influenza, parainfluenza virus, rhinovirus, adenovirus [22–25]	No studies	No studies	No studies	Animal and human studies- influenza [25–29]
Interferon	Immunomodulatory action leading to antiviral state	Potent antiviral effects seen [30, 31].	Effective in inhibiting cytopathogenic effects [32].	No studies	Effective in inhibiting SARS related CoV [33]	Not effective [34].	Animal model suggests benefit [35] but Clinical data does not [36, 37].	No studies	
Nitric oxide donor compounds*	Inhibits viral replication	Inhibits replication of SARS virus [38]	No studies	No studies	Japanese encephalitis [39] and flaviviruses [40]	InhaledNO improved arterial in patients with SARS [41]	No studies	No Studies	Decreased severity of Coxsackie myocarditis [42]
Oseltamavir	Neuraminidase inhibitor	Not effective [31]	No studies	No studies	Influenza	No studies	No studies	No studies	Influenza
Teicoplanin	Inhibits viral entry via by inhibiting the enzymatic action of Cathepsin L [43].	Blocks viral entry [43]	Blocks viral entry [43]	Blocks viral entry [44].	Ebola [43], HCV [45], Flaviviruses, Influenza, HIV [46–48]	No studies	No studies	No studies	No studies

*Includes inhaled NO, S-Nitroso N acetyl penicillamine, Glycyrrhizin

### Anti-parasitic drugs

#### Chloroquine (CQ) and hydroxychloroquine (HCQ)

Chloroquine (CQ) is a synthetic form of quinine (derived from the bark of cinchona tree) and is widely used as an anti-malarial since the last seventy years. Hydroxychloroquine (HCQ) has an extra hydroxyl group at the end of the side chain and is commonly used in the management of lupus and rheumatoid arthritis. Both these drugs have shown to have some anti-viral properties and may be useful in treating patients with COVID-19. Both CQ and HCQ interfere with the glycosylation of ACE-2 receptor, which is essential for the viral entry [49, 50]. Both the drugs are a weak base, and they interfere with the acidification of lysosome. This interferes with the pH-dependent endosome mediated viral entry [49, 50]. Both the drugs inhibit activation of cells by MAP kinase (P38 MAP kinase [49, 50] and inhibit post-translational modification of M proteins, thereby altering viral assembly and budding [49, 50]. Also, both the drugs are immunomodulatory agents and reduce pro-inflammatory cytokines [49, 50]. Compared to CQ, HCQ has a better in-vitro potency (7.6 times more potent), safety profile and lesser drug-drug interactions. HCQs have high accumulation in cells and long elimination half-life.

CQ has shown activity against various viruses in-vitro including HIV, hepatitis A/B/C, influenza A/B, dengue, chikungunya, Nipah, Hendra, Lassa and Ebola [51–53]. In-vitro data also suggests that CQ can inhibit coronaviruses (SARS-CoV-1, MERS CoV and Human corona OC43) also [54–56]. Recent studies have shown that CQ is also active in-vitro against SARS-CoV-2 [57]. CQ and HCQ decreased viral replication of SARS-CoV-2 in a concentration dependant manner. HCQ exhibits superior in-vitro anti-viral effect in comparison to CQ when the drug is added before viral challenge [58]. In-vitro synergistic effect of HCQ and azithromycin has been demonstrated in a recent study [59].

CQ has some activity in mice against human coronavirus OC43 [60]. Both CQ and HCQ reach up to 700 times higher level in lungs than in plasma [50]. According to pharmacology based pharmacokinetic modelling by Yao et al., simulated lung, blood and plasma concentration of CQ increased slowly after the first dose was given and was yet to achieve steady-state on Day 10. However, in HCQ, the concentration increased rapidly and reached a steady-state following the initial loading dose and subsequent maintenance dose [58].

Although in-vitro studies demonstrate the activity of CQ against SARS-CoV-2, this does not guarantee simultaneous in-vivo activity. For example, CQ was found to be effective in inhibiting replication of dengue, chikungunya and influenza in-vitro, but failed to show similar effects in in-vivo studies [60–62]. Preliminary reports from China in which 100 patients were given CQ showed early defervescence of fever and improvement in radiological findings. No serious adverse events were noted [63]. A French clinical trial of 36 PCR confirmed patients showed that virological clearance on Day 6 was significantly higher in HCQ arm compared to the control group (Table 1) [3]. In another study of 62 patients (31- standard treatment, 31- additional HCQ) with pneumonia associated with COVID-19 from China, additional HCQ for 5 days resulted in earlier remission of fever and cough [64]. Patients with severe/ critical illness were, however, excluded from the study. In a multi-centric open labelled randomized controlled trial of 150 patients from China, there was no difference in virological conversion rate or improvement in clinical symptoms at day 10. However, the HCQ arm showed a better clinical response in posthoc analysis when the effect of other anti-antivirals was removed (Table 1) [4]. A small French study of 11 patients by Molina et al. failed to show beneficial effects (early clearance of virus) of combining HCQ and azithromycin in patients with COVID-19 [65]. In another multi-centric retrospective study of 181 patients with COVID pneumonia from France, there was no difference in worse clinical outcomes between the two arms (Table 1) [5]. In a quasi-randomized controlled trial by Barbosa et al., of the 63 recruited patients, 32 received HCQ while 31 received standard support. Higher respiratory support requirement was noted in the HCQ group after 5 days of therapy [66]. In a retrospective study on 368 veterans from the United States of America, risk of death was found to be higher in those patients who received HCQ alone compared to no HCQ. No difference in requirement of ventilation was found between HCQ and no HCQ group (Table 1) [6].

The dosing recommendation, according to modelling by Yao et al. recommends- Day 1–400 mg twice daily and day 2–5- 200 mg twice daily [58]. However, the French group used a dosing regimen of 200 mg thrice daily for 10 days [3]. Drug-drug interactions and co-morbidities (pregnancy, chronic renal impairment) should be considered while defining the doses. Administration with food may be helpful as the bioavailability is increased with food. Although some experts are recommending routine use of CQ and HCQ as prophylaxis, there is no evidencing supporting this recommendation. CQ and HCQs have been successfully used for malarial prophylaxis, but similar results have not been observed for viral infections [61]. CQ has a narrow therapeutic window, but when appropriate dosing is used, it is relatively well tolerated. Compared to CQ, HCQs are better tolerated. Minor side effects include diarrhoea, nausea, vomiting and pruritus. Toxic doses may lead to life-threatening cardiomyopathy, macular retinopathy and neurotoxicity.

#### Nitazoxanide

Nitazoxanide is an oral anti-parasitic drug that is active against several protozoans, cestodes, helminths. It exerts its anti-parasitic activity by inhibiting pyruvate-ferredoxin oxidoreductase (PFOR), an essential enzyme in anaerobic energy metabolism [67]. Recently, laboratory studies have suggested its role as a broad-spectrum antiviral agent [68]. In influenza, it inhibits the maturation of the viral hemagglutinin, whereas it interferes with viral morphogenesis in rotavirus [68]. It can also limit virus entry, viral release and cell-to-cell transmission. It can also interfere with host-regulated pathways and can inhibit/ suppress the production of pro-inflammatory cytokines, including IL-6 and TNF alpha [69, 70].

Nitazoxanide has been shown to inhibit SARS-CoV-2 in in-vitro studies [57, 71]. In an in-vivo study based on a mouse model, nitazoxanide was found to markedly lower plasma IL-6 levels [70]. In a clinical trial conducted by Gamino-Arroyo et al., nitazoxanide did not show any difference when compared to placebo in patients with influenza [72]. The same trial included 17 cases of coronavirus and did not show any effect of nitazoxanide on the outcome.

Although in-vitro studies indicate that there might be a potential role of nitazoxanide in management of COVID-19, there is no clear evidence that it might be useful in the clinical setting. It is generally well-tolerated, and side effects include gastrointestinal disturbances, transaminitis, elevated creatinine and enlarged salivary glands [67].

#### Niclosamide

Niclosamide is a chlorinated salicylamide used for the treatment of infection with trematodes [73]. It inhibits ATP production by uncoupling oxidative phosphorylation in the mitochondria of the parasite [74]. It is also known to have antiviral effects by blocking the endosomal acidification [73, 75]. Endosomal acidification is important for the fusion of the viral envelope protein with the host membrane [76–78].

Niclosamide inhibits replication of various viruses in-vitro including influenza, dengue, chikungunya virus, Ebola virus and Hepatitis C [75–77]. It also inhibits replication of SARS and MERS viruses [79, 80]. Niclosamide has not been evaluated yet for the treatment of viral infections (coronavirus) in animal models or clinical studies.

Niclosamide has potential activity against coronaviruses based on in vitro studies alone, but there is a lack of data for the efficacy of this drug on SARS-CoV-2. Use of niclosamide is associated with mild and infrequent side effects that include gastrointestinal disturbances, malaise, pruritus and lightheadedness.

#### Ivermectin

Ivermectin is a broad-spectrum anti-parasitic drug used commonly in the treatment of strongyloidiasis and onchocerciasis [81]. It acts by inhibiting nuclear transport activity. The in-vitro activity has been demonstrated against several viruses including HIV, dengue, West Nile virus and influenza. Recently, it has also been found to inhibit SARS-CoV-2 in cell cultures [82]. In-vivo studies for the use of ivermectin as an antiviral are scarce. In a clinical trial for the use of ivermectin in dengue, it reduced levels of NS1 antigen but had no impact on viral load and clinical outcomes [83]. It is a relatively safe drug with good tolerability [81].

### Protease inhibitors

#### Lopinavir/ritonavir (LPV/r)

Lopinavir is a protease inhibitor used commonly for the treatment of HIV 1 infection. Ritonavir is used for boosting the lopinavir levels as a combination in sub-therapeutic doses for its inhibitory action on CYP3A4. In HIV, LPV inhibits the protease enzyme (aspartic protease family), thereby preventing the cleavage of Gag-Pol protein precursors. This results in the formation of immature and non-infectious virions. It is postulated that LPV has similar action on SARS-CoV-1 by inhibiting the chymotrypsin-like protease.

LPV/r has in-vitro activity against SARS-CoV-1 and MERS CoV [32, 84]. In another study, the addition of LPV/r to IFN B in MERS CoV infected cell lines did not enhance the activity of IFN B alone [85]. LPV/r was also found to have activity against Human coronavirus 229 E [32]. No in-vitro data on SARS-CoV-2 has been reported as of now. In humanized transgenic mice, LPV/r plus IFN B was not able to reduce the viral load of MERS CoV [85]. However, in non-human primate models (marmosets) infected with MERS CoV, LPV/r was able to reduce viral load and improve clinical progress [86]. In a study on health care workers exposed to MERS, lower rates of infection were noticed when they were given post-exposure prophylaxis of ribavirin and lopinavir/ritonavir for 14 days [87]. In patients of SARS-CoV-1 without ARDS, the addition of LPV/r to ribavirin and corticosteroids resulted in better clinical outcomes when compared to historical controls who received ribavirin and corticosteroids [84]. In a retrospective matched cohort study, LPV/r resulted in better clinical outcomes in patient with SARS [88]. Small case series have reported apparent benefit with LPV/r. Ten patients with COVID-19 were given sustained LPV/r with good outcomes [16]. In another study, 17 patients who received oral LPV/r alone, 52.9% of the patients showed clearance of viraemia on day 14 [16]. However, an open labelled trial of 199 COVID-19 patients did not show any decrease in time to clinical improvement, mortality or viral load after addition of LPV/r (Table 1) [7]. Although this trial failed to show any benefit of LPV/r, it can be argued that patients had already developed lung injury at the time of enrolment. The median time of enrolment in this study was 13 days [7]. It is postulated that since the viraemia is present in the early part of the illness and the lung involvement is a result of cytokine release and immune response, the drug may be effective when given in the early part of the illness. In the study by Chan et al. on patients with SARS-CoV-1, LPV/r was beneficial when given in the early part of illness but did not have any significant impact when it was given as a salvage or rescue [88].

A dose of 400/100 twice daily for up to 14 days has been tried in most studies. It has to be kept in mind that this drug has several drug-drug interactions and may require dose modification in pregnancy. No dose modification is required in patients with kidney disease or mild hepatic impairment. It is recommended to avoid LPV/r in patients with severe hepatic impairment (Child-Pugh C or Alanine transaminase >5X Upper limit of normal). Gastrointestinal side effects and hypertriglyceridemia are common in patients on LPV/r. Peripheral lipoatrophy and visceral adiposity are also noticed in patients on long-term LPV/r. Serious adverse events include pancreatitis, hepatotoxicity and QT prolongation.

#### Simeprevir and Paritaprevir

Simeprevir and paritaprevir are oral NS3/4A protease (chymotrypsin-like protease) inhibitors that are used for the treatment of chronic hepatitis C [89]. Chymotrypsin like protease is also present in SARS CoV-2, which is essential to cleave an 800-kDa polypeptide to generate various proteins [90]. There are no in-vitro or in-vivo studies available that have assessed the role of these drugs in coronavirus infections. However, molecular docking analysis studies indicate simeprevir and paritaprevir could fit well to the binding pocket of protease [90, 91]. The safety profile of both drugs is generally acceptable. Use of simeprevir is associated with hyperbilirubinemia [89].

### Polymerase inhibitor

#### Remdesivir

Remdesivir is an adenosine analogue that binds to RNA-dependent RNA polymerase. It gets incorporated into nascent viral RNA chains resulting in its premature termination.

Replication inhibition has been demonstrated in a wide range of viruses in vitro and in vivo [57, 85, 92–97]. The therapeutic efficacy of remdesivir was first described in an animal model against Ebola. Subsequently, Dyer et al. described preliminary findings suggesting mortality benefit when remdesivir was given in the early stages of Ebola [94]. The drug has exhibited in vitro and in-vivo activity against SARS-CoV-1 and MERS-CoV [85, 92, 96]. Wang et al. showed that the use of remdesivir is effective against SARS-CoV-2 in Vero E6 cell lines [57]. Case reports of success with remdesivir in patients with COVID-19 have been documented [98, 99]. In a recent multi-centric single-arm study by Grein et al., remdesivir was used in 53 patients of COVID-19, improvement in oxygen support class was demonstrated in 68% of the patients (Table 1) [8]. This drug has a long half-life and needs once-daily dosage. The trial by Grein et al. used dosing of 200 mg on day one, followed by 100 mg from day 2 to day 10 [8]. The most common side effects are transient gastrointestinal symptoms and transaminitis.

#### Favipiravir

Favipiravir is a promising antiviral drug which targets the viral RNA-dependent RNA polymerase [100]. The drug gets converted to its active form by host enzymes and has exhibited considerable activity in influenza [100]. In a small clinical trial of COVID-19 patients, when compared to another potential drug arbidol, it showed a faster clinical recovery rate at day seven and more effectively reduced incidence of fever and cough [101]. In an open labelled trial of 70 patients in China, favipiravir had better viral clearance and improved lung imaging when compared to lopinavir/ritonavir [102]. The dosing used in the study was 1600 mg twice daily on the first day and 600 mg twice daily on day 2 to day 14. Side effects include raised serum uric acid levels, psychiatric symptoms and gastrointestinal disturbances [101].

#### Nucleoside analogues

Sofosbuvir is an oral nucleoside analogue (NS5B polymerase inhibitor) that is used for the treatment of Hepatitis C [103]. Invitro studies have shown its effectiveness in SARS CoV 1. Molecular docking studies have shown that sofosbuvir may be active against COVID-19 [103].

Galidesivir is another broad-spectrum antiviral drug that has exhibited efficacy against Ebola and yellow fever [104].

Ribavirin is used for the treatment of hepatitis C and viral hemorrhagic fevers. The in-vivo studies in SARS-CoV-1 and in-vitro studies in SARS-CoV-2 have not been encouraging. Besides, usual clinical dosing is associated with haematological toxicity.

### Fusion inhibitors

Nafamostat mesylate and Camostat mesylate are drugs which are used to treat acute pancreatitis. These drugs act by effectively blocking the membrane fusion between the viral envelope and host cell plasma membrane. The in-vitro activity has been demonstrated for MERS-CoV [105]. Similarly, enfuvirtide and SC29EK are fusion inhibitors used for the treatment of HIV [106, 107]. These drugs have been suggested to have a possible role in SARS-CoV-2.

### Monoclonal antibodies

#### Tocilizumab

Tocilizumab is a humanized monoclonal antibody that is used in several rheumatological conditions like rheumatoid arthritis, juvenile idiopathic arthritis, Castleman’s disease, giant cell arteritis and cytokine release syndrome caused by CAR-T treatment [108]. Tocilizumab acts against the soluble and membrane-bound IL-6 receptors and inhibits the inflammatory action of IL-6 [108]. Pulmonary injury and ARDS has been postulated to be a result of hyper-inflammatory state in the latter half of the illness in COVID-19 due to increase in levels of pro-inflammatory cytokines such as IL-6 [109, 110]. Similar studies from patients with influenza have shown that high IL-6 levels are associated with severity [111]. However, mice models show that IL-6 is also useful to prevent virus-induced neutrophil death and is a useful host response in early infection. Therefore, it is imperative to understand that molecules such as tocilizumab can only be used only in patients with severe disease when cytokine release syndrome is suspected as evidenced by an increased IL-6. There are no published reports of the use of tocilizumab in SARS and MERS. Preliminary case series have shown good outcomes with tocilizumab (400 mg) in 21 patients with severe or critical COVID-19. All the patients either improved (19/21) or were improving (2/21) at the time of reporting [112]. In another study of 30 patients, it was found that the use of tocilizumab was associated with lesser ICU admission and requirement of mechanical ventilation when compared to controls (Table 1) [9].

Tocilizumab is given as an intravenous infusion over 1 hour at a dose of 8 mg/kg in patients who weigh more than 30 kg (maximum dose- 800 mg). This can be repeated for three additional times, 8 hours apart. Adverse reactions include upper respiratory tract infections, headache and transaminitis [108]. In COVID-19 clinical studies, tocilizumab was shown to be safe except for a few reports of transaminitis [9].

#### Siltuximab

Siltuximab is another chimeric antibody that blocks the effect of IL-6 and is used for the treatment of multicentric Castleman’s disease. In a single-arm trial of 21 patients with COVID and ARDS from Italy, 33% of the patients improved, and 43% of the patients remained stable on treatment [113].

#### Meplazumab

The spike protein of SARS-CoV-2 binds to CD 147 of the host cell during entry. Meplazumab is a humanized monoclonal antibody that acts against CD147 and therefore has a potential role in the management of COVID-19. In a small open labelled trial from China (*n* = 28), use of intravenous meplazumab (*n* = 17) was associated with better virological (time to negativity) and clinical outcomes (lesser severity and earlier discharge) when compared to controls (*n* = 11) [114]. No adverse effects were reported with meplazumab.

### Miscellaneous drugs

Several other drugs with anti-viral properties are being re-purposed for use in COVID-19. The details of the following drugs have been tabulated in Table 2: alisporivir, arbidol, auranofin, doxycycline, isprinosine, interferon, nitric oxide compounds, oseltamivir and teicoplanin.

## Conclusion

The data on these drugs are increasing every day. It is tempting to try these drugs in the name of safety or ease of availability. Some initial studies showed benefit with HCQ in early viral clearance. Subsequent studies failed to show such benefit. Lopinavir/ritonavir was shown to be ineffective when started late in patients with COVID pneumonia. However, their effectiveness in early COVID is debatable. Initial results of compassionate use of remdesivir are encouraging but the drug needs to be evaluated in well-designed randomized trials before it can be used routinely. Tocilizumab has been proposed for use in severe or life-threatening cases of cytokine release syndrome based on studies with very small sample size which have shown good results. Till the time, the data on these drugs come from well conducted clinical trials are available, judicious and well-informed use is the need of the hour.

## Data Availability

Not applicable.
